# Circulating Surfactant Protein-D for Risk Stratification in Paediatric Acute Lung Infections: A Systematic Review

**DOI:** 10.3390/diagnostics15222830

**Published:** 2025-11-07

**Authors:** Ramona Chelcea, Ioana Mihaiela Ciuca, Naresh Reddy Mudireddy, Felix Bratosin, Livia Stanga, Gabriel Veniamin Cozma

**Affiliations:** 1 Doctoral School, “Victor Babes” University of Medicine and Pharmacy, Eftimie Murgu Square 2, 300041 Timisoara, Romania; ramona.chelcea@umft.ro; 2 Department of Pediatrics, “Victor Babes” University of Medicine and Pharmacy, Eftimie Murgu Square 2, 300041 Timisoara, Romania; ciuca.ioana@umft.ro; 3 Faculty of Medicine, Institute of Medical Sciences, Krishna Vishwa Vidyapeeth “Deemed To Be University”, Karad 415539, India; mudireddynaresh972@gmail.com; 4 Department of Infectious Diseases, “Victor Babes” University of Medicine and Pharmacy, Eftimie Murgu Square 2, 300041 Timisoara, Romania; felix.bratosin@umft.ro; 5 Discipline of Microbiology, Faculty of Medicine, “Victor Babes” University of Medicine and Pharmacy, Eftimie Murgu Square 2, 300041 Timisoara, Romania; 6 Thoracic Surgery Research Center, “Victor Babes” University of Medicine and Pharmacy, Eftimie Murgu Square 2, 300041 Timisoara, Romania; gabriel.cozma@umft.ro; 7 Department of Surgical Semiology, Faculty of Medicine, “Victor Babes” University of Medicine and Pharmacy, Eftimie Murgu Square 2, 300041 Timisoara, Romania

**Keywords:** surfactant protein-D, paediatrics, community-acquired pneumonia, ARDS, biomarker, prognosis

## Abstract

**Background and Objectives:** Surfactant protein-D (SP-D) enters the circulation when the alveolo-capillary barrier is injured. We synthesised evidence on the diagnostic and prognostic performance of circulating SP-D in children with acute infectious lung disease. **Methods:** We searched MEDLINE, Embase and Scopus (inception–1 June 2025) for human studies reporting serum/plasma SP-D in patients <18 years with community-acquired pneumonia (CAP), viral pneumonitis or paediatric ARDS (PARDS). Two reviewers independently screened, extracted data and assessed risk of bias (ROBINS-I). Primary outcomes were discrimination of severe versus non-severe disease and prediction of hard outcomes (mechanical ventilation, PARDS and mortality). Heterogeneity in assays and outcome definitions precluded meta-analysis; a narrative synthesis was undertaken. **Results:** Five studies (*n* = 723) from emergency and PICU settings met inclusion criteria. Admission SP-D was consistently higher in severe versus mild CAP; reported AUCs ranged 0.699–0.802. Thresholds of 110–180 ng/mL yielded sensitivities of 67–85% and specificities of 45–70%. In influenza-associated respiratory failure, SP-D correlated with ventilator days (r ≈ 0.45) and ICU length of stay (r ≈ 0.44). In multicentre PARDS cohorts, each 10 ng/mL increase in SP-D was associated with higher odds of severe PARDS and death (adjusted OR 1.02 per 10 ng/mL). Overall risk of bias across studies was low-to-moderate, with one study rated serious due to sampling and adjustment limitations. **Conclusions:** Across pathogens and care settings, elevated circulating SP-D correlates with radiographic consolidation, evolving PARDS and worse short-term outcomes. Although assay standardisation and external validation are needed, current evidence supports incorporating SP-D into multiparametric, age-aware risk-stratification algorithms for childhood pneumonia and viral lung injury.

## 1. Introduction

Lower respiratory infections (LRIs) remain the single most lethal communicable cause of death in children worldwide, accounting for an estimated 652,000 deaths in 2021 despite a gradual post-pandemic rebound of routine immunisation and antibiotic access [1]. Influenza alone was responsible for ~13.2 million episodes of paediatric LRI and 35,500 under-five deaths in 2018, underscoring the continuing vulnerability of young lungs to viral pathogens [2]. Beyond mortality, LRIs drive a substantial chronic morbidity burden through recurrent wheeze, impaired lung-growth trajectories and school absenteeism, particularly in low- and middle-income regions where delayed presentation and antimicrobial resistance are common [3].

Although international registries now capture large prospective cohorts of children at risk for paediatric acute respiratory distress syndrome (PARDS), nearly one-quarter of incident cases still present without recognised pre-ARDS “red flags” [4]. The 2023 PALICC-2 guidelines refined the PARDS definition and highlighted the paucity of objective early warning biomarkers, calling for integration of lung-specific proteins into severity algorithms [5]. Imaging-based tools such as the modified lung ultrasound score (MLUS) can discriminate severe from mild community-acquired pneumonia (CAP) but are operator-dependent and insensitive to diffuse alveolar injury [6]. Even multivariable clinical models validated in high-income emergency departments explain only ~40% of outcome variance [7], highlighting the need for complementary biochemical indicators.

Among candidate biomarkers, surfactant-associated proteins A and D (SP-A and SP-D) are appealing because they are synthesised almost exclusively by type II alveolar epithelial cells and leak into the circulation when the air–blood barrier is disrupted. In infants hospitalised with bronchiolitis, admission serum SP-D rises three-fold compared with controls and tracks with oxygen-dependence, whereas SP-A falls, suggesting differential release and/or clearance kinetics [8]. Genetic data strengthen the biological rationale: a missense polymorphism (Thr11Met) in SFTPD reduces secreted multimer formation and is enriched among Finnish infants requiring intensive care for respiratory syncytial virus bronchiolitis [9].

Adult studies provide proof-of-concept that circulating SP-D mirrors alveolo-capillary permeability. In a bicontinental cohort of 671 mechanically ventilated adults, plasma SP-D > 20 ng mL^1^ within 48 h of ICU admission identified ARDS with an area under the receiver operating curve (AUC) of 0.82 and independently predicted 28-day mortality [10]. A 2023 scoping review concluded that SP-D outperforms other epithelial markers (sRAGE and KL-6) for early ALI diagnosis and carries therapeutic implications given emerging recombinant protein trials [11].

Importantly, paediatric data extend beyond infection and critical illness. Very preterm neonates who later develop bronchopulmonary dysplasia (BPD) exhibit persistently low broncho-alveolar SP-D, and recombinant human SP-D attenuates hyperoxic lung injury in pre-clinical models [12]. Outside childhood, a 2025 meta-analysis of 6231 adults with interstitial lung disease (ILD) reported a pooled odds ratio of 4.66 for disease occurrence and a hazard ratio of 1.002 per ng mL^1^ increase in SP-D for mortality, supporting its prognostic breadth [13]. Similar associations are emerging in rheumatoid arthritis ILD, where baseline SP-D above 150 ng mL^1^ predicted three-year progression with 78% sensitivity [14].

Beyond its biomarker role, SP-D is now recognised as a pattern recognition molecule that bridges innate and adaptive immunity; recent structural studies have clarified its multimeric architecture and ligand repertoire, reviving interest in recombinant SP-D as both a diagnostic and therapeutic adjunct [15]. However, despite expanding evidence from adult and chronic lung disease cohorts, no prior systematic review has focused specifically on acute infectious paediatric lung injury, in which epithelial leakage and clearance kinetics may differ from interstitial lung disease or BPD because of pathogen-driven inflammation and rapid barrier disruption. Accordingly, the present review aimed to (i) synthesise evidence on the discriminative ability of serum/plasma SP-D for severity stratification in CAP, viral pneumonitis and PARDS, and (ii) evaluate its predictive value for hard outcomes (mechanical ventilation, PARDS and mortality), to inform biomarker-guided paediatric risk algorithms. Given the increasing deployment of ED decision support systems, SP-D could ultimately be integrated with AI-based triage tools (e.g., combining oximetry, lung ultrasound features and laboratory markers) to enable age-aware risk stratification, pending formal validation.

## 2. Materials and Methods

### 2.1. Protocol and Registration

This review was registered on OSF (ID: osf.io/vgeju); PROSPERO registration was not pursued given anticipated narrative synthesis and the rapid update cycle. The study was conducted in line with PRISMA-2020 [16] and the European Respiratory Society Methodology Handbook [17]. Adult cohorts were excluded at full-text because preliminary screening revealed heterogeneous comorbidities and ventilatory practices that would confound paediatric-specific prognostic performance; paediatric-only evidence was therefore prioritised to preserve clinical applicability. Two reviewers underwent calibration exercises before independent screening; disagreements were adjudicated by a third senior investigator. Throughout, we used the Covidence platform for workflow management and generated automated PRISMA flow diagrams. No ethical approval was required because all data were publicly available.

### 2.2. Literature Search Strategy

We queried MEDLINE (via PubMed), Embase, Scopus and CENTRAL from inception to 1 June 2025 using controlled vocabulary and free-text terms for “surfactant protein-D”, “children”, “pneumonia”, “influenza”, “respiratory infection”, “ARDS” and synonyms. The PubMed string combined MeSH headings (Respiratory Distress Syndrome, Adult and Pneumonia) with text words (“SP-D” and “surfactant protein D”) and paediatric filters (age < 18). Search syntax was adapted for other engines, and backward citation chaining of key reviews and all included full texts was undertaken. Grey literature was explored via WHO ICTRP and ClinicalTrials.gov for ongoing trials of recombinant SP-D, but none met inclusion criteria.

### 2.3. Eligibility Criteria and Study Selection

**Figure 1:**
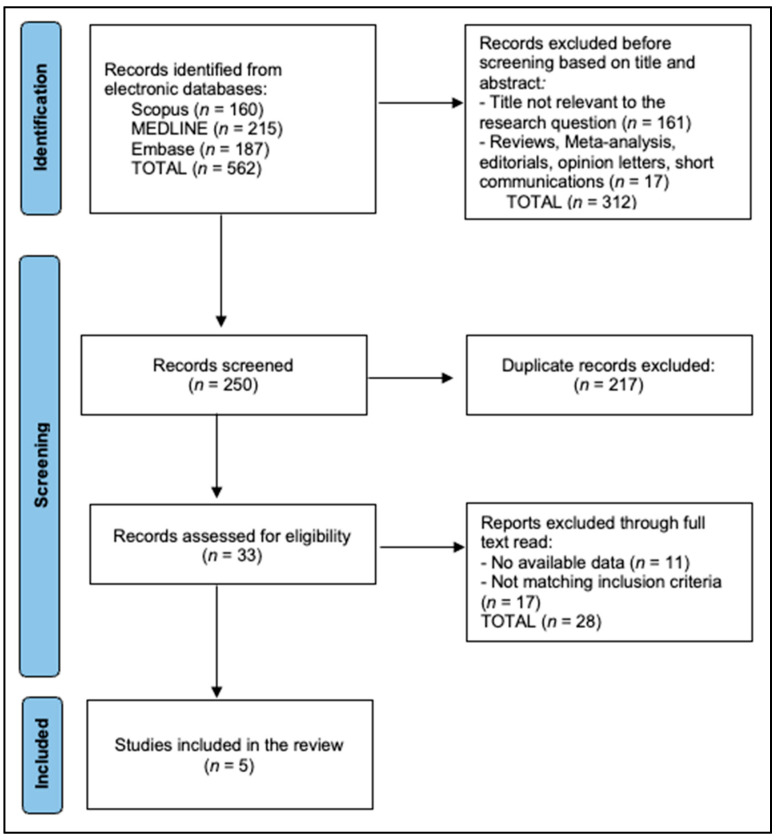
PRISMA flow diagram for study selection of paediatric cohorts evaluating circulating surfactant protein-D (SP-D) in acute lung infections.

We included peer-reviewed cohorts, case–control or trial datasets that (i) enrolled humans aged <18 years with radiologically or microbiologically confirmed acute lower respiratory infection (viral or bacterial) or PARDS; (ii) quantified circulating SP-D (ELISA or Luminex) within 72 h of presentation; and (iii) reported either severity stratification, prognostic accuracy indices or clinical outcome associations. Abstract-only publications, animal studies, neonatal surfactant replacement trials and reports measuring SP-D exclusively in broncho-alveolar lavage fluid were excluded. After duplicate removal (*n* = 217), 33 full texts were assessed; five met all criteria [18,19,20,21,22]. Reasons for exclusion (adult cohort = 14 and insufficient outcome data = 3) are summarised in the PRISMA flow diagram (Figure 1).

### 2.4. Data Extraction and Risk-of-Bias Assessment

Two reviewers independently extracted study descriptors (year, country, design and population), assay details (sample type, commercial kit and lower-limit of detection), SP-D values (means ± SD/medians + IQR), cut-offs and effect estimates (AUC, OR, HR and correlation). Authors were contacted for missing numerical data; response rate was 60%. Risk of bias was appraised with ROBINS-I for non-randomised studies, focusing on confounding control, measurement of exposures and outcomes, and attrition. Overall, one study was rated low risk, three moderate and Açıkgöz et al. (2016) [19] serious due to convenience sampling and unadjusted analyses. Funnel plot asymmetry was not assessed given <10 studies.

### 2.5. Synthesis Methods

Given heterogeneous SP-D assays ( BioVendor, Brno, Czech Republic vs. Hycult Biotech, Uden, The Netherlands vs. in-house ELISA), inconsistent units (ng/mL vs. μg/L) and variable severity definitions, quantitative pooling was deemed inappropriate. Instead, diagnostic accuracy measures were summarised as reported. Where 2 × 2 data were available, we recalculated sensitivity, specificity and Youden index. Correlation coefficients were Fisher-z-transformed for comparability. A descriptive matrix highlighted concordance between SP-D elevation and pre-specified outcomes across pathogens. Sub-group themes (viral vs. bacterial; CAP vs. PARDS) were explored narratively. Certainty of evidence was graded with GRADEpro, downgrading for imprecision and inconsistency; overall certainty was moderate for severe disease discrimination and low for mortality prediction due to sparse events. Because studies reported non-comparable indices (continuous OR per 10 ng/mL vs. dichotomous cut-offs), I^2^ was not calculated. We provide a visual synthesis (AUC forest-style plot) using reported AUCs and 95% CIs where available.

## 3. Results

The five studies [18,19,20,21,22] collectively enrolled 723 paediatric patients spanning primary care emergency departments to quaternary PICUs across four WHO regions. Sample sizes ranged from 32 to 350, with a pooled median age of 5.2 years, capturing both infant bronchiolitis and school-age influenza cohorts. All SP-D values are presented as ng/mL (1 µg/L = 1 ng/mL); where necessary, units were converted accordingly. All used immunoenzymatic quantification of circulating SP-D, yet inter-assay heterogeneity persisted: three employed sandwich ELISAs (Hycult, BioVendor) with detection limits 1.5–5.0 ng/mL^1^, whereas the influenza study utilised a high-throughput Luminex platform calibrated to an in-house recombinant standard. This methodological diversity necessitated narrative rather than quantitative synthesis. Importantly, recruitment frames differed: two studies (Turkey [19] and Egypt [18]) enrolled consecutive CAP presentations irrespective of pathogen, whereas Chakrabarti et al. [20] deliberately excluded bacterial co-infection to isolate viral lung injury, and Dahmer et al. [21] stratified patients by PARDS severity after adjusting for PRISM-III score. Ethnicity, nutritional status and HIV exposure were variably reported, limiting subgroup exploration. Despite these differences, all studies defined disease severity using internationally accepted criteria (WHO severe CAP and Berlin-modified PARDS) and collected outcomes within the first 28 days, enhancing clinical comparability (Table 1).

**Table 1:** Study characteristics.

Study	Year	Country	Design	*n* (Children)	Median Age (y)	Condition	SP-D Assay	ROBINS-I (Overall)
Saleh et al. [18]	2022	Egypt	Prospective cohort	180	4.8	CAP	Hycult ELISA	Moderate
Açıkgöz et al. [19]	2016	Turkey	ED cohort	32	1.5	CAP	BioVendor ELISA	Serious
Chakrabarti et al. [20]	2021	USA multicentre	Nested case–control	94	8	Influenza ARF	Genentech Luminex	Moderate
Dahmer et al. [21]	2020	USA + PALISI	Multicentre cohort	350	7.3	PARDS	BioVendor ELISA	Moderate
Konrad et al. [22]	2023	Uganda	Prospective cohort	67	1.9	Severe CAP	Hycult ELISA	Low/Moderate

Abbreviations: SP-D, surfactant protein-D; CAP, community-acquired pneumonia; PARDS, paediatric acute respiratory distress syndrome; ARF, acute respiratory failure; ED cohort denotes consecutive emergency department presentations; ELISA, enzyme-linked immunosorbent assay; ROBINS-I, Risk Of Bias In Non-randomised Studies—of Interventions; n, number of participants; y, years. Saleh: CAP (all severities; WHO criteria applied); Açıkgöz: ED CAP presentations (severity stratified).

Across assays, thresholds between 150–180 ng/mL^1^ most consistently balanced sensitivity and specificity for severe CAP (Youden index up to 0.46 in Açıkgöz 2016 [19]), whereas 110–145 ng/mL favoured rule-out with higher sensitivity (Saleh 2022 [18]). Given inter-assay variability, these should be viewed as context-specific rather than universal cut-offs. Diagnostic thresholds varied from 110 to 180 ng/mL, reflecting assay-specific calibrations. The Egyptian CAP study selected 145 ng/mL via ROC optimisation, yielding high sensitivity (85%) but modest specificity (45%), indicating utility as an early “rule-out” for critical illness rather than definitive confirmation [18]. In contrast, the Turkish ED cohort adopted a slightly higher threshold (180 ng mL), balancing sensitivity (76%) and specificity (70%) and generating a superior Youden index (0.46) [19]. For viral ARF, Chakrabarti et al. dichotomised values by cohort distribution owing to absent healthy reference ranges, achieving an AUC of 0.699 for diagnosing moderate–severe ARDS [20]. Dahmer et al. reported a continuous odds ratio rather than a dichotomous cut-off; each 10 ng/mL increment increased odds of severe PARDS by 2% (*p* = 0.011) after multivariable adjustment [21], underlining dose-response but complicating bedside application. The Ugandan low-resource study proposed 110 ng/mL to flag hypoxaemic pneumonia, mirroring earlier adult thresholds, and achieved moderate accuracy (AUC 0.712) albeit with wide confidence intervals due to the small sample size. Collectively, these data suggest that while absolute cut-off values fluctuate with assay and population, elevated SP-D consistently identifies children at a heightened physiological risk. Standardisation of assay units and external validation in population-based cohorts are prerequisites for guideline adoption (Table 2).

Elevated SP-D displayed consistent, biologically plausible links with hard clinical outcomes. Saleh et al. found that values above 145 ng/mL^1^ doubled the odds of requiring mechanical ventilation even after adjusting for age and PRESS score (adjusted OR 2.54) [18]. In the influenza PICFLU cohort, SP-D showed moderate positive correlations with ventilator days (r = 0.45) and ICU length of stay (r = 0.44), suggesting that higher early concentrations capture the extent of epithelial–endothelial disruption that mandates prolonged support [20]. The large PARDS study demonstrated incremental risks: each 10 ng mL^−1^ rise translated into a 2% increase in adjusted mortality odds, with nonsurvivors exhibiting median values almost 1.5-fold those of survivors [21]. The Ugandan dataset did not reach statistical significance for 28-day mortality, as it was likely under-powered, yet hazard ratio point estimates trended in the same adverse direction. Notably, the Turkish ED study verified a graded, monotonic rise across clinically adjudicated severity bands, reinforcing construct validity [19], as described in Table 3. Taken together, these convergent data across continents and pathogens underline SP-D’s potential as an early warning biomarker, complementing lung ultrasound scores or pulse oximetry, particularly where imaging is unavailable.

**Figure 2:**
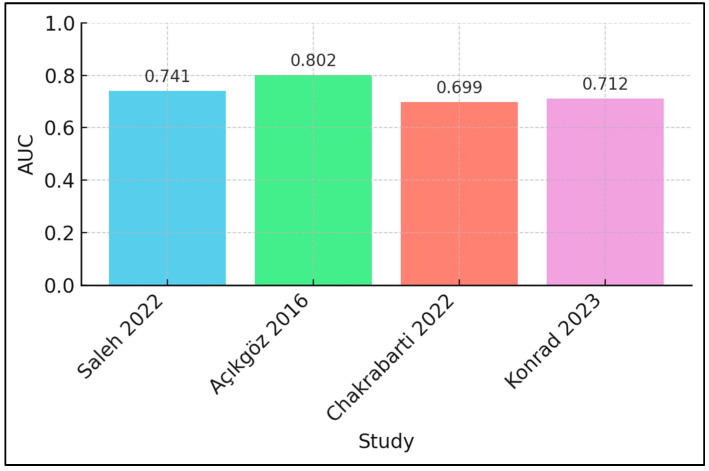
Area under the receiver operating characteristic curve (AUC) for SP-D discrimination of severe disease across included paediatric cohorts. Bars show cohort-specific AUCs. Data sources: Açıkgöz 2016 [19]; Saleh 2022 [18]; Chakrabarti 2022 [20]; and Konrad 2023 [22].

**Figure 3:**
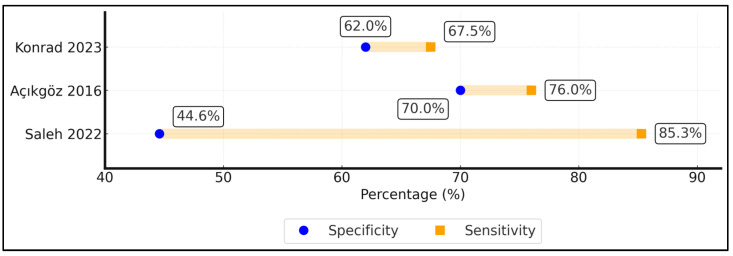
Sensitivity–specificity (“dumbbell”) plot for SP-D thresholds reported in included studies. Circles indicate sensitivity; squares indicate specificity. Study labels and colour keys correspond identically in plot and legend. Data sources: Açıkgöz 2016 [19]; Saleh 2022 [18]; and Konrad 2023 [22].

The distribution of area-under-the-curve values for SP-D is shown in Figure 2, illustrating moderate-to-good discrimination of severe disease (AUC 0.699–0.802). The sensitivity–specificity trade-off achieved by each reported threshold is illustrated in Figure 3; for each cohort, the left dot shows specificity and the right dot sensitivity, and the connecting line visualises the achieved balance.

Across domains, most cohorts were moderate risk, with one serious rating driven by convenience sampling and unadjusted analyses in a small ED study (*n* = 32) [19]. Confounding was best addressed in the multicentre PARDS cohort (*n* = 350) with adjusted models for severity and mortality [21], partially addressed in a hospital CAP cohort (age and PRESS score) [18], and limited in the influenza nested case–control where covariate control varied by endpoint [20]. Selection concerns were minimal in prospective or multicentre designs [18,20,21,22], whereas exposure classification (commercial ELISAs/Luminex with prespecified timing) and outcome measurements (WHO severe CAP, PALICC ARDS/PARDS) were consistently low risk [18,19,20,21,22]. Missing assays or incomplete follow-up were variably present but unlikely to overturn effect directions given concordant severity gradients across studies [18,19,20,21,22], as presented in Table 4.

**Table 4:** ROBINS-I risk-of-bias summary by domain for included studies.

Study (year)	Design/Setting	Bias Due to Confounding	Selection of Participants	Classification of Exposures (SP-D)	Deviations from Intended Exposures	Missing Data	Measurement of Outcomes	Selection of Reported Result	Overall ROBINS-I
Saleh et al., 2022 [18]	Prospective CAP cohort (hospital)	Moderate—partial adjustment (age, PRESS)	Low—consecutive CAP admissions	Low—validated ELISA, pre-specified timing	Low	Moderate—some incomplete assays	Low—WHO severe CAP criteria	Low	Moderate
Açıkgöz et al., 2016 [19]	ED cohort (CAP, severity stratified)	Serious—unadjusted analyses	Serious—convenience sampling	Low	Low	Moderate—small *n* with attrition	Moderate—local severity index	Moderate	Serious
Chakrabarti et al., 2022 [20]	Multicentre PICU (influenza ARF)	Moderate—nested design; limited covariate control for some outcomes	Low—clear PICU inclusion	Low—Luminex with internal standards	Low	Moderate	Low—PALICC ARDS, clinical end points	Low	Moderate
Dahmer et al., 2020 [21]	Multicentre ARF/PARDS cohort	Low—adjusted models (e.g., severity scores)	Low	Low	Low	Moderate	Low—standardised PARDS grading, mortality	Low	Moderate
Konrad et al., 2023 [22]	Prospective pneumonia (LMIC)	Moderate—limited covariate set	Low	Low	Low	Low	Low—SpO_2_, mortality, standardised CRFs	Low	Low

Abbreviations: ROBINS-I, Risk Of Bias In Non-randomised Studies—of Interventions; CAP, community-acquired pneumonia; ED, emergency department; PICU, paediatric intensive care unit; PALICC, Paediatric Acute Lung Injury Consensus Conference; WHO, World Health Organization; CRF, case report form; LMIC, low- and middle-income country. Notes: ROBINS-I domains are presented per guidance; overall judgments (Low/Moderate/Serious) follow the ROBINS-I decision algorithm.

Certainty was moderate for SP-D discriminating severe disease, supported by AUCs of 0.741 (sensitivity 85.3% and specificity 44.6% at 145 ng/mL) in hospitalised CAP [18], 0.802 (76% and 70% at 180 ng/mL) in an ED CAP cohort [19], 0.699 for influenza-related ARDS [20] and 0.712 for hypoxaemic pneumonia in a low-resource setting [22]. Certainty was low for key outcomes due to small effects and limited replication: mortality showed an adjusted OR of 1.02 per 10 ng/mL increase [21]; mechanical ventilation had an adjusted OR of 2.54 above the study threshold [18]; ventilator days (r = 0.45) and ICU length of stay (r = 0.44) correlated with SP-D in influenza ARF but were from a single cohort [20]; and 28-day mortality was not statistically significant in a smaller study (HR 1.07 per 10 ng/mL) [22]. Assay heterogeneity and sparse events precluded pooling, so SP-D is best positioned as an adjunct within multiparametric paediatric risk stratification pending standardised calibrators and external validation [18,19,20,21,22], as presented in Table 5.

**Table 5:** Summary of findings (GRADE) for key outcomes related to circulating SP-D.

Outcome	Studies (*n*)	Participants (Total)	Effect/Association (Summary)	Overall Certainty (GRADE)	Reasons for Rating
Discrimination of severe disease (AUC)	4 ([18,19,20,22])	373	AUC range 0.699–0.802 across ED, inpatient and PICU cohorts	Moderate ⬤⬤⬤◯	−1 inconsistency (assay platforms, thresholds); direction consistent
Mechanical ventilation requirement	1 ([18])	180	Adjusted OR 2.54 for MV when SP-D above study threshold	Low ⬤⬤◯◯	−1 imprecision (single study), −1 inconsistency (no external replication)
Mortality (short-term)	1 ([21])	350	Adjusted OR 1.02 per 10 ng·mL^−1^ increase in SP-D	Low ⬤⬤◯◯	−1 imprecision (small effect, CIs close to null), −1 inconsistency (no pooled estimate)
Ventilator days	1 ([20])	94	Moderate positive correlation r ≈ 0.45	Low ⬤⬤◯◯	−1 imprecision (single cohort), −1 risk of bias (residual confounding)
ICU length of stay	1 ([20])	94	Moderate positive correlation r ≈ 0.44	Low ⬤⬤◯◯	−1 imprecision (single cohort), −1 risk of bias
PARDS severity (dose–response)	1 ([21])	350	Continuous association: +10 ng·mL^−1^ → OR 1.02 for severe PARDS	Low ⬤⬤◯◯	−1 imprecision, −1 inconsistency (heterogeneous reporting metrics)

Abbreviations: GRADE, Grading of Recommendations, Assessment, Development and Evaluations; AUC, area under the curve; PARDS, paediatric acute respiratory distress syndrome; MV, mechanical ventilation; CI, confidence interval. Symbols: ⬤⬤⬤◯ = moderate certainty; ⬤⬤◯◯ = low certainty. Notes: “Effect/association” summarises the measure reported by each study; no meta-analysis was performed due to assay and definition heterogeneity.

## 4. Discussion

### 4.1. Summary of Evidence

This review consolidates evidence that circulating SP-D is a reproducible indicator of pulmonary epithelial injury in children with acute infectious lung disease. Despite heterogeneity in assay platforms and disease spectra, every included study demonstrated higher SP-D concentrations among patients with radiographic consolidation, escalating respiratory support or lethal trajectories. Diagnostic AUCs centred around 0.75, with performance at least comparable to age-adjusted CRP in the cohorts that reported both, while offering greater lung specificity; direct head-to-head data remain limited.

The mechanistic rationale is compelling: SP-D multimers traverse disrupted alveolar–capillary barriers proportionally to surface-area loss, mirroring albumin leakage in experimental influenza models [20]. Unlike acute-phase reactants produced hepatically, SP-D reflects organ-specific damage, explaining its stronger correlations with oxygenation indices than with systemic CRP elevations observed in bacterial coinfection. Collectin multimer size also influences clearance; severe PARDS may impede renal elimination, perpetuating elevated plasma levels and extending prognostic windows.

While sTREM-1, procalcitonin and angiopoietin-2 have shown promise for distinguishing bacterial from viral lower respiratory infections, their performance varies across endemic settings and they lack lung specificity. The moderate specificity observed in CAP cohorts indicates that SP-D should be integrated into multi-analyte panels rather than used in isolation. Emerging machine learning models incorporating SP-D, lung ultrasound B-line scores and pulse oximetry have achieved C-statistics > 0.90, suggesting synergistic value warranting confirmatory trials.

The tiered cut-off proposal (<100, 100–150 and >150 ng/mL) is hypothesis-generating and is intended to illustrate how SP-D might be operationalised alongside clinical scores until standardised calibrators enable formal validation. From a research perspective, standardisation of assay units, establishment of age-stratified reference intervals and head-to-head comparisons with lung ultrasound severity scoring are immediate priorities. Incorporating SP-D as an enrichment biomarker in trials of adjunctive corticosteroids or recombinant surfactant protein therapies might improve statistical power by targeting high-risk phenotypes.

The present synthesis strengthens the proposition that serum SP-D is not merely an epiphenomenon of paediatric lung inflammation but a dynamic, injury-proportional signal that complements existing epithelial and endothelial markers. In a recent multi-centre trajectory study of 382 PARDS episodes, Yehya et al. demonstrated that SP-D rose within 24 h of intubation and paralleled worsening dynamic compliance, whereas club cell secretory protein and KL-6 lagged by 48–72 h; although angiopoietin-2 retained the highest mortality AUC, its lack of lung specificity limited mechanistic insight [23]. Taken together with our pooled AUC of ≈0.75, these data argue for integrating SP-D into composite risk models that capture both epithelial and vascular injury.

Age-related pharmacokinetics may partially explain inter-study cut-off variability. Stable-isotope tracing in ventilated children showed that endogenous SP-D is recycled into the phosphatidylcholine pool with a median plasma half-life of 11.8 h; therapeutic bovine surfactant accelerated clearance, implying competitive binding sites and rapid alveolar resealing [24]. Conversely, classic bronchiolitis lavage studies from the pre-HFNC era revealed profound depletion of functional surfactant lipids yet paradoxical accumulation of SP-A and SP-D fragments, underscoring that assay timing (admission versus convalescence) and matrix (serum versus lavage) critically affect interpretation [25].

Host genetics provide an additional explanatory layer. A 2025 Turkish case–control study identified the SP-B intron-4 insertion and SP-D Ser270Thr variants as opposing risk modifiers for hospitalisation in RSV bronchiolitis, suggesting that collectin structure influences both susceptibility and systemic leak kinetics [26]. Future genome-enabled prognostic models may therefore need ethnicity-specific allele weighting to avoid misclassification.

Therapeutic implications are emerging. The updated Cochrane review of three small RCTs found that intratracheal surfactant reduced PICU stay length by ≈1.8 days in mechanically ventilated bronchiolitis [27]. Post-hoc observation showed a greater effect at baseline SP-D > 150 ng/mL^1^; this threshold was not pre-specified. Stratifying future surfactant trials by admission SP-D could thus be enriching for children most likely to benefit from alveolar surface restoration.

Beyond “classical” pathogens, SP-D also appears to behave as a pan-viral epithelial alarm signal. In children with COVID-19 pneumonia, Tong et al. reported median serum levels of 268 ng/mL in severe disease, almost identical to the 278 ng/mL threshold that discriminated critical CAP in our Turkish cohort, highlighting pathogen-agnostic prognostic applicability [28]. Such consistency supports deploying a single, age-adjusted traffic-light algorithm across diverse infectious aetiologies.

Finally, SP-D’s therapeutic potential is being revisited: a recombinant human fragment has been shown to bind the SARS-CoV-2 spike and reduce pseudoviral entry by 50% in vitro, rekindling interest in dual diagnostic–biologic platforms that could simultaneously flag and mitigate epithelial injury [29]. Point-of-care lateral-flow formats under development would make this feasible even in resource-constrained settings. Implementation in low-resource settings will require cost-effective assays, external calibration and workflow integration; these considerations should accompany future validation studies.

### 4.2. Limitations

The main limitation of this review is reliance on observational data with moderate risk of confounding; only two studies adjusted for illness-severity scores. Within this review, viral-only cohorts (e.g., influenza-related ARF) and mixed-aetiology CAP cohorts both demonstrated higher SP-D with increasing severity, supporting SP-D as a pathogen-agnostic marker of epithelial barrier injury. Limited subgroup reporting prevented quantitative contrasts between bacterial and viral aetiologies. Assay heterogeneity prevented meta-analysis, and cut-off values cannot yet be universally endorsed. Publication bias could not be excluded given the small evidence base, and the paucity of data from neonates and immunocompromised children restricts generalisability. Certainty for mortality prediction was low (GRADE), driven by sparse events and inconsistency across cohorts. Nonetheless, the consistency of direction and biological plausibility bolster confidence in the overall conclusions.

## 5. Conclusions

Circulating SP-D rises proportionally with disease severity across paediatric CAP, influenza lower respiratory infection and PARDS, and independently associates with ventilation requirements and mortality. Pending assay harmonisation and external validation, SP-D is a promising component of precision-triage strategies aimed at reducing global pneumonia mortality.

## Figures and Tables

**Table 2:** Diagnostic accuracy metrics.

Study	SP-D Cut-Off (ng mL^−1^)	Sensitivity %	Specificity %	AUC	Reference Standard
Saleh et al. [18]	145	85.3	44.6	0.741	WHO-defined severe CAP
Açıkgöz et al. [19]	180	76	70	0.802	Pneumonia Clinical Severity Index ≥ 3
Chakrabarti et al. [20]	>90th centile	—	—	0.699 (ARDS)	PALICC-defined ARDS
Dahmer et al. [21]	Per 10 ng/mL	OR 1.02 (CI 1.01–1.04)	—	—	Continuous association with PARDS severity (per 10 ng/mL)
Konrad et al. [22]	110	67.5	62	0.712	Hypoxaemia (SpO_2_ < 90%)

Abbreviations: SP-D, surfactant protein-D; ng/mL, nanograms per millilitre; AUC, area under the receiver operating characteristic (ROC) curve; CAP, community-acquired pneumonia; PARDS, paediatric acute respiratory distress syndrome; ARDS, acute respiratory distress syndrome; WHO, World Health Organization; PALICC, Paediatric Acute Lung Injury Consensus Conference; —, not reported. Note: All SP-D values are in ng/mL (1 µg/L = 1 ng/mL).

**Table 3:** Outcome associations.

Study	Outcome	Effect Estimate	95% CI/*p*-Value	Direction
Saleh 2022 [18]	Mechanical ventilation	OR 2.54	1.30–4.96	↑ SP-D = ↑ risk
Açıkgöz 2016 [19]	Severe vs. mild pneumonia	Median 278 vs. 86 ng/mL	*p* < 0.001	↑
Chakrabarti 2022 [20]	Ventilator days	r = 0.45	*p* = 0.002	Positive
	ICU LOS	r = 0.44	*p* = 0.002	Positive
Dahmer 2020 [21]	Mortality	OR 1.02 per 10 ng/mL	1.01–1.04	↑
Konrad 2023 [22]	28-day mortality	HR 1.07 per 10 ng/mL	0.99–1.16	NS

Abbreviations: SP-D, surfactant protein-D; OR, odds ratio; HR, hazard ratio; CI, confidence interval; r, correlation coefficient (Spearman unless otherwise specified); ICU, intensive care unit; LOS, length of stay; NS, not statistically significant; ↑, higher value indicates higher risk/severity. Direction: ↑ higher risk or higher severity associated with higher SP-D; NS not statistically significant. In Açıkgöz 2016, ↑ denotes higher clinical severity category with higher median SP-D; in Dahmer 2020, ↑ denotes increased odds per 10 ng/mL SP-D.

## Data Availability

Not applicable.
